# The Effect of Hydroxyl Moieties and Their Oxosubstitution on Bile Acid Association Studied in Floating Monolayers

**DOI:** 10.1155/2014/152972

**Published:** 2014-12-25

**Authors:** Márta Szekeres, Béla Viskolcz, Mihalj Poša, János Csanádi, Dušan Škorić, Erzsébet Illés, Ildikó Y. Tóth, Etelka Tombácz

**Affiliations:** ^1^Department of Physical Chemistry and Materials Science, University of Szeged, Aradi Vt. 1, Szeged 6720, Hungary; ^2^Department of Chemistry and Chemical Informatics, University of Szeged, Boldogasszony Sugárút 6, Szeged 6720, Hungary; ^3^Laboratory of Physical Pharmacy, Department of Pharmacy, Faculty of Medicine, University of Novi Sad, Hajduk Veljkova 3, 21000 Novi Sad, Serbia; ^4^Department of Chemistry, Faculty of Sciences, University of Novi Sad, Trg Dositeja Obradovica 3, 21000 Novi Sad, Serbia

## Abstract

Bile salt aggregates are promising candidates for drug delivery vehicles due to their unique fat-solubilizing ability. However, the toxicity of bile salts increases with improving fat-solubilizing capability and so an optimal combination of efficient solubilization and low toxicity is necessary. To improve hydrophilicity (and decrease toxicity), we substituted hydroxyl groups of several natural bile acid (BA) molecules for oxogroups and studied their intrinsic molecular association behavior. Here we present the comparative Langmuir trough study of the two-dimensional (2D) association behavior of eight natural BAs and four oxoderivatives (traditionally called keto-derivatives) floated on an aqueous subphase. The series of BAs and derivatives showed systematic changes in the shape of the compression isotherms. Two types of association could be distinguished: the first transition was assigned to the formation of dimers through H-bonding and the second to the hydrophobic aggregation of BA dimers. Hydrophobic association of BA molecules in the films is linked to the ability of forming H-bonded dimers. Both H-bond formation and hydrophobic association weakened with increasing number of hydroxyl groups, decreasing distance between hydroxyl groups, and increasing oxosubstitution. The results also show that the Langmuir trough method is extremely useful in selecting appropriate BA molecules to design drug delivery systems.

## 1. Introduction

The basic molecule of bile acids (BAs) and derivatives is cholan-24-oic acid (cholanic acid, CnA). Its bended steroidal skeleton is hydrophobic and the carboxylic group at the end of the molecule renders the molecule slightly amphiphilic. Bile acids are the derivatives of cholanic acid, in which −OH substituents are oriented toward the concave plane of the skeleton, similarly to the orientation of the carboxylic moiety. As a result, bile acids are amphiphilic with respect to the molecular plane. The planar amphiphilicity can be modified by altering the type, number, location, and orientation of the hydrophilic substituents. The salts of bile acids and derivatives are intended to apply as drug delivery vehicles, membrane permeability, and drug absorption enhancers [1–5] because of their unique structural properties. Their association is characterized by relatively high values of critical micelle formation concentration (CMC) and by the simultaneous formation of dimers, tetramers, and higher associates [3, 6, 7]. Bile salts preferably form mixed micelles with one or more hydrophobic molecules [3]; for example, fats are solubilized in vivo in bile acid-cholesterol mixed micelles. The mechanism of association of bile salts in aqueous environment is not fully understood. Based on the wide variety of experimental and theoretical methods, two basic views have developed. One set of experimental and theoretical results assures that the primary associates are due to hydrophobic interactions, from which secondary micelles and higher aggregates can form via H-bonding [8–10]. According to another set of results [3, 11], the primary association is due to H-bonding; and further aggregation occurs via hydrophobic association of the dimers that are hydrophobic from the outside supported also in modelling studies by coauthors of the present work [12]. NMR spectroscopy studies by Funasaki et al. [13] suggest that the extent to which H-bonding is involved in dimer formation depends largely on the molecular structure of bile salts. Heat capacity change in bile salt demicellization measured by isothermal titration calorimetry [14] also shows that large part of hydrophobic surfaces in the micellar structure must be accessible by water and points to H-bonding possibility between the molecules.

The membranolytic activity of bile salts limits their biomedical application: natural bile salts with 1–3 OH-substituents at different positions on the steroidal skeleton dissolve cell membranes and solubilize cell membrane fatty acids [15]. On the other hand, replacement of hydroxy substituents with oxogroups leads to a different incorporation of BAs in the cell membrane. The oxocompounds generated from a bile acid are traditionally called keto-derivatives or keto acids. The brain uptake of several drugs was enhanced by applying monoketocholic acid [16], the mechanism of which is that the oxo-BA increases the elasticity of the membrane and promotes both the active and the passive transport of drug molecules. Owing to the general interest in designing biocompatible BA derivatives for biomedical applications, many other types of derivatives are reported in literature as well, such as amino acid and sugar substituted bile acids [17, 18]. In the present work, we investigate the changes in the association behavior of BA derivatives in Langmuir films caused by the variation of the number and location of oxogroups in the steroidal skeleton. One of our primary motivations for the use of Langmuir monolayers was to attempt to study the intrinsic association properties of bile acids free from interfering solvent effects.

The interaction between amphiphilic molecules is frequently characterized in two dimensions by spreading and compressing insoluble monolayers on aqueous surfaces in Langmuir trough experiments [19–23]. This method has the advantage that it excludes the most of solvent-solute interactions (all except the hydration of hydrophilic groups) and the molecular interactions can be studied more directly as compared to three-dimensional solution experiments. The compression isotherms indicate clearly the expanded or condensed states of the films, phase transitions (the coexistence of molecular and aggregated domains), and area demands of individual molecules at specific surface pressure values. Thermodynamic and different other theoretical approaches have been applied to analyze the pressure-area diagrams (the compression isotherms) and to evaluate the phase behavior of stable films [24–28]. Mixed films are prepared to study the interaction between dissimilar molecules with the aim of predicting, for example, how drug molecules are incorporated into or transported through lipid bilayers [29–31].

Langmuir trough studies of some of the most common bile acids can be found in literature [8, 32–41]. One of the general conclusions is that the shape of the isotherms strongly depends on the subphase pH. Flat plateau regions have been observed in several isotherms and assigned to BA film collapse. The structure of bile acids can be systematically modified by removal or substitution of the OH-groups in the steroid skeleton. Up to now, only randomly selected BAs and derivatives have been studied by the Langmuir trough method; however, this work provides a study of the effect of systematic changes in the molecular structure of BAs on their two-dimensional association. The whole series of 11 studied BAs comprises nonsubstituted (CnA), monosubstituted (lithocholic acid, LCA), disubstituted (deoxycholic acid, DCA, and derivatives chenodeoxycholic, ursodeoxycholic, and hyodeoxycholic acids, CDCA, UDCA, and HDCA, resp.), and trisubstituted (cholic acid, CA, and dehydroderivatives, 3DHDCA, DHDCA, 3DHCA, and DHCA) cholanic acids. The substituents in each of the molecules are either hydroxy- or oxogroups or both hydroxy- and oxogroups. Our aim was to establish the order of relative association propensity of the studied BAs and derivatives and to find correlation with their molecular structure. We attempted to assign the changes in the slopes of the compression isotherms to the transitions of molecular ordering in the films. Analysis of the isotherms allowed us to derive molecular interaction energy parameters for both H-bond formation and hydrophobic interaction and, in addition, to get a new insight into their relative roles in BA association.

## 2. Experimental

### 2.1. Materials

**Figure 1:**
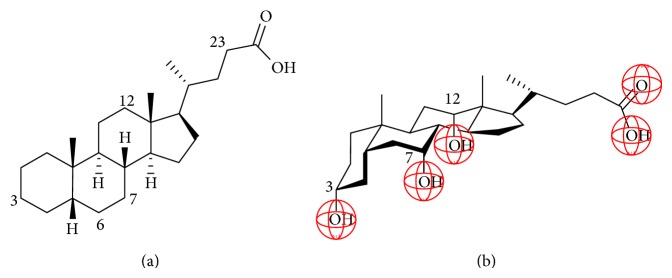
The structure of cholanic acid (CnA, (a)) and the steric presentation of cholic acid (CA, (b)).

5*β*-Cholanoic acid (Figure 1(a)), the basic molecule of cholic acid (Figure 1(b)) and derivatives, was synthesized from dehydrocholic acid (DHCA) by the Wolff-Kishner procedure [42]. Cholic acid (CA) and deoxycholic acid (DCA) were purchased from Alfa Easer, Germany (the purity was 99.98%). Hyocholic acid (HCA), chenodeoxycholic acid (CDCA), hyodeoxycholic acid (HDCA), ursodeoxycholic acid (UDCA), and lithocholic acid (LCA) were purchased from Sigma, New Zeeland (the purity was 99.98%). The oxoderivatives were prepared from the parent CA or DCA molecules by dehydrogenation. 3-Dehydrocholic acid (3DHCA) and 3-dehydrodeoxycholic acid (3DHDCA) were synthesized by the oxidation (equivalent to dehydrogenation) of the hydroxyl group at C3 position according to the method of Tserng [43]. Fully oxidized (dehydrogenized) dehydrocholic acid (DHCA) and dehydrodeoxycholic acid (DHDCA) were prepared according to the procedure of Fieser and Rajagopalan [44]. The synthesis products were subjected to chromatographic purification (the purity was 99%). All the studied bile acids (BAs) and their systematic names are collected in Table 1. In this paper we use the naming rules according to the recommendations of the IUPAC-IUB Joint Commission on Biochemical Nomenclature (JCBN) [45] and the propositions of Hofmann et al. [46]. Methanol, NaCl, and HCl were products of analytical purity from Molar, Hungary. Ultrapure water was used in the experiments from MilliQ RG system (Merck Millipore, Billerica, MA, USA).

### 2.2. Langmuir Trough Experiments

The monolayers were spread from methanolic solutions of BAs on aqueous subphases in a Kibron *μ*TroughXS (Kibron Inc., Helsinki, Finland) Langmuir trough. Methanol was used to prepare BA solutions, because the more hydrophilic BAs (e.g., DHCA) could not be dissolved in apolar solvents such as chloroform, hexane, or cyclohexane. The concentration of the BAs was ~0.1 mg/mL. 40–60 *μ*L of the solutions was applied to spread ~9 nmoles of molecules on the aqueous surface with resulting surface density of 200–300 Å^2^/molecule. A small diameter (0.51 mm) special metal alloy wire was used to measure surface pressure (resolution is 0.2 mg and sensitivity is better than 0.01 mN/m) yielding excellent reproducibility of the isotherms. The BA films were compressed at a speed of 6.5 mm^2^/min. The inaccuracy in the film area was less than 0.5%. The experiments were conducted at constant temperature 25 ± 0.1°C controlled by a thermostat and the temperature control plate of Kibron and repeated at least three times to check the reproducibility. Temperature dependence was measured for LCA and DCA at 15, 25, 35, and 45°C and negligible effect on the isotherms was found. The optimal composition of the aqueous subphase was determined in film-dissolution experiments. Various subphase solutions were coated by a talcum powder layer and a drop of BA solution in methanol was applied to the surface, subsequently. The clearing of the talcum from the middle of the aqueous surface revealed the formation of the BA film. The decrease in the film area in time was measured by using the area analyzing software JMicroVision 1.2.7. A composition of 3 M NaCl solution at pH = 1.2 was found to prevent quick dissolution of BA films in agreement with the published suggestions of 3–5 M NaCl and pH = 1–3 [8, 35, 36, 38–41]. The observed kinetics of dissolution was used to determine the maximum allowable time of the film age during the compression measurements.

## 3. Result and Discussion

### 3.1. Solubility of BA Films in Aqueous Subphase

It is well known that bile acids and derivatives are significantly soluble in water and their solubility increases with the amount and hydrophilicity of hydrophilic substituents and with pH. The aqueous solubility and 3D aqueous aggregation data of the studied BA molecules are shown in Table 1. The general trend is that with increasing solubility the CMC also increases. Despite the absence of solubility data for all the oxoderivatives in literature, it is intuitive that replacing an increasing number of OH-substituents by oxogroups enhances the solubility and CMC. In case of the dihydroxy- and trihydroxy-BAs, a relatively wide range of CMC values has been found depending on the method. For the mixed oxohydroxy derivatives and the trioxo-derivative data in the literature are very rare; thus we can rely only on the CMC values published by us earlier [47] reflecting significant decrease in the aggregation propensity (i.e., increase in solubility) of BAs in water with increasing number of oxogroups.

**Figure 2:**
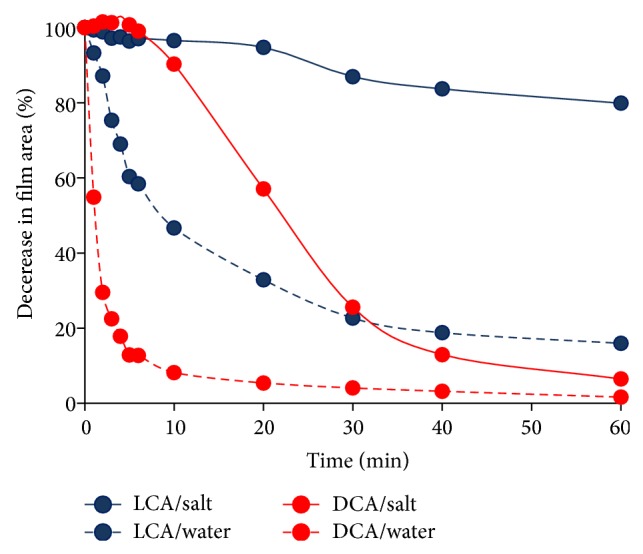
Decrease in the area of the LCA and DCA films with time on the surface of pure water and salt (3 M NaCl, pH = 1.2) subphase.

For the Langmuir trough experiments, it is important to assure the best possible stability of the films, that is, to prevent their dissolution in the subphase. The solubility of bile acids and derivatives cannot be fully eliminated; nevertheless, we performed preliminary experiments in order to decrease their dissolution by limiting the duration of the Langmuir trough measurements. In addition to subphase composition optimization (see in Section 2.2 Langmuir trough experiments), dissolution kinetics experiments were performed to optimize the time frame for the film compression experiments. The results for the DCA and LCA films spread on the surface of 3 M NaCl solution at pH = 1.2 and pure water are presented in Figure 2. A movie file made from the pictures of film areas taken at particular intervals within 60 minutes is also provided as Supplementary Material (in Supplementary Material available online at http://dx.doi.org/10.1155/2014/152972). The film of the most hydrophobic molecule LCA was stable up to 20 minutes and the moderately hydrophilic DCA up to 5 minutes on the subphase of 3 M NaCl at pH = 1.2 (lower left and right films in the movie). At the same time, both films dissolved rapidly in pure water (upper films in the movie). The Langmuir trough experiments were designed on the basis of the above observations. Instead of the commonly accepted 10–20 minutes we applied 2 minutes for the film equilibration and solvent evaporation similarly to the CnA compression isotherms of Ekwall and coworkers [35]. Although solvent evaporation may be incomplete, the probable effect of the remaining methanol is the same in all BA films and so it does not influence the relative association behavior of the BA molecules. The films were fully compressed within 17 minutes using a compression speed of 11 cm/min (65 mm^2^/min). The age of the films thus was less than 20 minutes in all cases. During that period the LCA film was essentially stable and the DCA film also remained at the interface in ~60% (Figure 2). Due to the inherent aqueous solubility of BAs, the compression isotherms do not reflect the behavior of a stable film and their thermodynamic analysis is not plausible. It is well demonstrated, however, that the two-dimensional association properties of the BAs affect the shape of the isotherms [8, 35, 36, 38–41] and so meaningful conclusions regarding the molecular interactions can be deduced.

### 3.2. Compression Isotherms

The isotherms were measured at low subphase pH value to prevent the dissociation of carboxylic groups and at high salt concentration to further reduce solubility of the acids. Nevertheless, all the molecules except for CnA and LCA dissolved to some extent, which must be taken into account during the evaluation. In general, the shape of the isotherms is determined by the interaction between the molecules in the monolayer and by the solvation of hydrophilic groups in the aqueous subphase.

**Figure 3:**
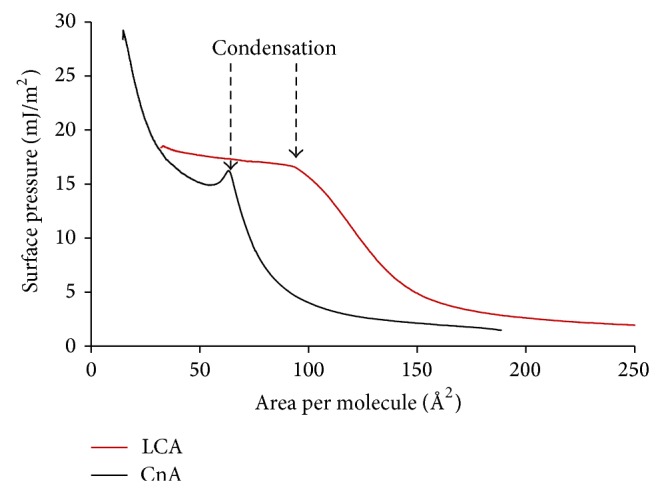
Compression isotherms of CnA and LCA measured on 3 M NaCl at pH = 1.2 aqueous subphase at 25 ± 0.1°C. Surface pressure unit is expressed in mJ/m^2^ instead of the commonly applied mN/m.

**Figure 4:**
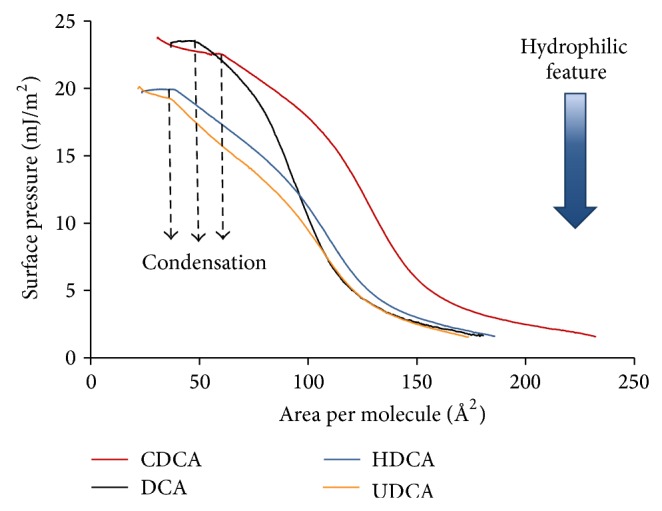
Compression isotherms of CDCA, DCA, HDCA, and UDCA measured on 3 M NaCl at pH = 1.2 aqueous subphase at 25 ± 0.1°C. Increasing hydrophilicity of molecules is indicated by the arrow. Surface pressure unit is expressed in mJ/m^2^ instead of the commonly applied mN/m.

**Figure 5:**
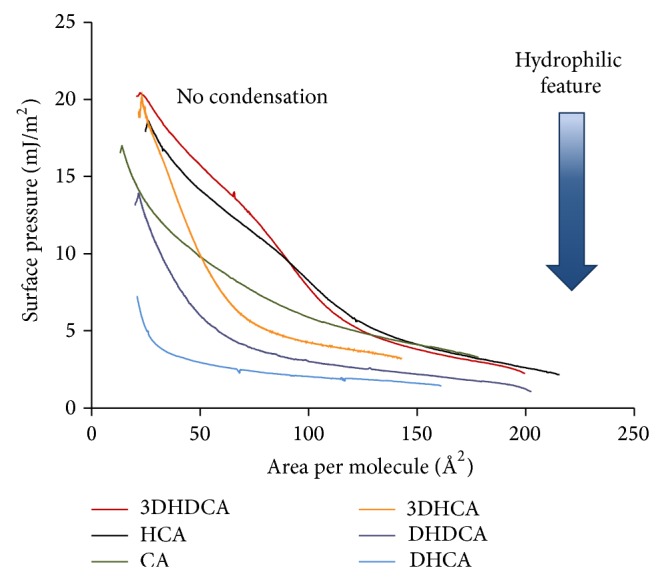
Compression isotherms of 3DHDCA, HCA, CA, 3DHCA, DHDCA, and DHCA measured on 3 M NaCl at pH = 1.2 aqueous subphase at 25 ± 0.1°C. Increasing hydrophilicity of BAs is indicated by the arrow. Surface pressure unit is expressed in mJ/m^2^ instead of the commonly applied mN/m.

Compression isotherms of the BA films are presented in Figures 3–5. At the beginning of compression, a gradual increase in surface pressure can be seen in all the isotherms, which is typical to liquid expanded films [23]. The phase transition between gaseous and liquid expanded states occurs at very low—near zero—surface pressure [51]. In our BA films, the surface pressure at spreading was near or above 1 mN/m and, consequently, the gaseous state was not observed. Another common feature of all isotherms is the definite collapse of the films seen at the lowest molecular area values as a sharp decrease in the surface pressure. The only exception is DHCA (Figure 5), for which the collapse could not be observed.

The main feature of the isotherms in Figures 3 and 4 is the presence of a flat region commonly assigned to first-order phase transitions. The plateau represents the coexistence of two different phases of the same material in a dynamic equilibrium, either two different monolayers (e.g., liquid expanded and liquid condensed) or a monolayer and its collapsed three-dimensional structure [52]. We should note that at longer equilibration (5–10 min) the plateau could not be reached, which shows the tendency of BAs to dissolve in the subphase after spreading. The isotherm of CnA (Figure 3) is special in that, instead of a flat condensation region, an overshoot in surface pressure can be seen at the first-order transition and that the film can be compressed to higher surface pressure values. The same type of the CnA isotherm was found by Ekwall et al. [36]. The principally different behavior of CnA monolayers compared to the other BAs is probably due to its high hydrophobicity and low amphiphilicity.

The condensation of the films starts at molecular area values of 100–40 Å^2^/molecules, decreasing in the order of LCA > CDCA > DCA > HDCA > UDCA > CnA. The molecular area at condensation reflects the interaction potential between the amphiphiles and between the hydrophilic groups of the amphiphiles and subphase water. The closer can the molecules be pressed to each other without condensation, the larger is their resistance to condensation. Well before condensation, an inflection is observed in the isotherms (except for the CnA film) that can be attributed to surface pressure-induced interfacial tilting of the molecules [37]. At the end of this orientational ordering region the slope becomes constant, resembling a second-order phase transition. It is likely that the adjacent tilted molecules can undergo face-to-face dimerization (depicted for solution phase bile salt aggregation by Funasaki et al. [13]) through H-bonding between the OH-groups which emerged from the subphase. As the H-bonded dimers are more hydrophobic than the monomers, the films become more and more stable in the course of dimer formation and withstand further compression before condensation begins. The response of the films to compression depends strongly on the number, position, and orientation of the –OH substituents. The LCA film condenses shortly after dimerization at relatively high molecular area and low surface pressure values. The length of the linear second-order phase transition region of the deoxycholic acid variations DCA, CDCA, HDCA, and UDCA increases gradually in parallel with their increasing hydrophilicity. Consequently, condensation of the latter BAs occurs at lower molecular area and higher surface pressure values.

LCA is the most hydrophobic BA, since it has only one −OH group at the 3C end of the molecule, opposite to the carboxylic end. The intermediate hydrophobic section of the molecules can be responsible for hydrophobic aggregation. The three distinct parts in its compression isotherm (Figure 3) are the liquid expanded phase region at large molecular area values, the inflection point in the liquid expanded region at 119 Å^2^/molecule, and the phase transition region from liquid expanded to liquid condensed phase [51] starting at 97 Å^2^/molecule. The film collapses 33 Å^2^/molecule without formation of a stable continuous liquid condensed phase. This is in harmony with the general observation [8] that “bile acids appear to form gaseous and liquid monolayers but not condensed or solid films.” LCA isotherms of principally similar shape were found by Small [8], Ekwall et al. [36], and Gálvez-Ruiz and Cabrerizo-Vílchez [38]; but there is no general agreement whether the plateau region is due to condensation or collapse of the film. Kellner and Cadenhead [53] studied the compression isotherms of hydroxyhexadecanoic acids (HHA) with the OH group attached at different carbon atoms of the chain and the results can be compared to that of LCA. All molecules exhibited a phase transition region of the compression isotherms and the 16HHA film did not reach the stable liquid condensed state similarly to that found in the LCA film. The structure of the 16HHA molecule resembles LCA in that the OH group is located at one end of the molecule opposite to the carboxylic end. The observation that the behavior of the LCA film is very similar to that of 16HHA suggests that the plateau of the LCA isotherm corresponds to condensation, rather than to collapse. The plateau region represents the coexistence of the liquid expanded and liquid condensed phases; but as the condensed phase becomes coherent, the film collapses instantly due to its rigidity. The collapse is observed as a definite abrupt decrease in the surface pressure. The majority of the analyses of LCA isotherms in literature ignore the marked change in the slope of the isotherms, that is, the inflection point prior to condensation (119 Å^2^/molecule for LCA). Kauffmann and coauthors [37], however, attributed the slope change in several BA compression isotherms to a second-order phase transition. Bonosi and coworkers [54] assigned the slope change in the isotherms to changing the orientation of the molecules at the interface studying the Langmuir monolayers of polyazamacrocycle derivatives. We suppose that similar orientational ordering can occur in the LCA film within the molecular area range 119 Å^2^/molecule (inflection) to 97 Å^2^/molecule (beginning of condensation). The value of 97 Å^2^/molecule is close to the molecular area of BAs lying flat in the interface (60–80 Å^2^ as calculated from the molecular geometry). At this state, forced tilting of LCA is possible with reducing film area, which can be followed by H-bond formation between some of the OH-groups leaving the aqueous subphase. The value of the molecular area at collapse (33 Å^2^/molecule) is close to the area requirement of the LCA molecules oriented perpendicularly to the interface (the minimum projection area is 35–45 Å^2^ based on molecular models [35] and from molecular geometry 7–14 Å^2^ can be calculated).

Contrary to LCA, the CnA film can be compressed to high surface pressure values after condensation (Figure 3). The condensation begins at ~60 Å^2^/molecule and leads to the formation of a coherent liquid condensed phase, which collapses at 14.8 Å^2^/molecule. This feature can be explained by the absence of the second anchoring hydrophilic group, allowing the molecules to tilt already at the start of the compression with the carboxylic group anchored to the aqueous phase and the steroidal skeleton directed into the air. The change in the slope and the linear part of the isotherm prior to the condensation are fully absent indicating that the orientational ordering occurs probably already at spreading. It is interesting to note that, as soon as the condensation begins, the surface pressure starts to decrease. This can be the result of efficient aggregation leaving CnA-free aqueous surface domains with higher surface tension, that is, smaller surface pressure.

The compression isotherms of deoxycholic acid variants CDCA, DCA, HDCA, and UDCA (Figure 4) share several common features, in line with their very similar chemical structure. They all have two OH-groups on the steroid skeleton, one of them at C3 and the other at different locations (C12 in DCA, C7 in CDCA, and C6 in HDCA) or same location but with different orientation (7*α*-hydroxy in CDCA and 7*β*-hydroxy in UDCA). At high molecular areas, a liquid expanded state is seen, in which the inflection point is observed reflecting the change in orientational ordering, just like in the LCA isotherm discussed above. At certain point of the isotherms, the slope becomes constant (resembling second-order phase transition) up to the start of condensation. After the relatively short condensation region a definite collapse occurs at specific molecular areas; thus, a stable condensed phase cannot develop. As for the LCA films, rigidity is obvious. The films of CDCA and DCA begin to condense at significantly higher surface pressure compared to HDCA and UDCA (~24 compared to ~20 mJ/m^2^). This is most likely connected with the increased solubility of the latter molecules. We note that according to data in [45, 46] (Table 1) UDCA is the least soluble among these molecules, which is not supported by our Langmuir film experiments. On the other hand, the CMC values in Table 1 increase in the order of increasing film solubility in harmony with our results. The linear part of the isotherms shows that the orientation of the molecules does not change within that region. We can suppose that the molecules which are lying flat at the air/water interface in the liquid expanded state start to tilt gradually with respect to the interface at the inflection point (128, 95, 110, and 108 Å^2^/molecule for CDCA, DCA, HDCA, and UDCA, resp.) and do not change their orientation until condensation begins. The molecular areas at the beginning of condensation (60, 49, 39, and 37 Å^2^/molecule for CDCA, DCA, HDCA, and UDCA, resp.) are considerably smaller than that required for a lying flat orientation (60–80 Å^2^/molecule). Once some of the OH-groups lose their contact with water at the inflection point of the isotherms, they become prone to H-bonding with another OH group from a neighboring BA molecule. In this way, dimers can form that are less hydrophilic than the monomers; and they can withstand higher compressional force without sinking into the subphase compared to their monomers. The molecular area values at the collapse points are similar to that of LCA but decrease significantly with solubility in the order of CDCA, DCA, HDCA, and UDCA (30.7, 37, 24.6, and 22 Å^2^/molecule, resp.). Condensation of these molecules reveals their comparatively high hydrophobicity that allows hydrophobic aggregation of the H-bonded dimers under constraint.

The remaining six BAs can also be grouped based on the similarity of their compression isotherms (Figure 5). The condensation region is absent in all isotherms. The films collapse in the liquid expanded state with the exception of DHCA, which does not collapse at all. In addition, the change in the slope of the isotherms can only be observed for 3DHDCA and HCA that are the least hydrophilic molecules of the series. Some indication of the slope change is also seen in the isotherm of CA. Except for 3DHDCA, all the molecules are modifications of the trihydroxy CA and the isotherms reflect their higher solubility as compared to the modifications of the dihydroxy molecule DCA (Figure 4). With increasing solubility, the tendency of the molecules to escape into the aqueous subphase during compression also increases. Thus, the absence of condensation regions in the isotherms reflects that hydrophobicity of these molecules is insufficient to reach hydrophobic association under constraint. Nevertheless, another type of association, that is, H-bond formation, is still possible in the case of those BAs with −OH substituents. However, the latter becomes fully hindered by dissolution in the subphase in the case of 3DHCA. The highest solubility of 3DHCA, DHDCA, and DHCA is also supported by the high CMC values in Table 1 (98, 51, and 130 mM, resp.). Considerable solubility of the molecules makes the area per molecule values irrelevant.

### 3.3. Two-Dimensional Molecular Interaction of BAs

**Figure 6:**
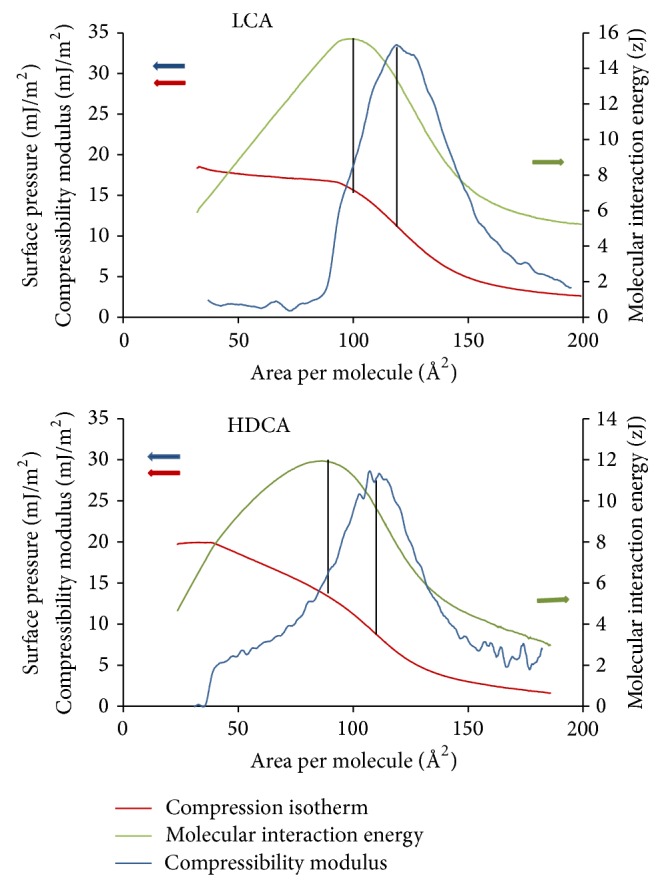
Compression isotherms (3 M NaCl, pH = 1.2 subphase at 25°C), compressibility modulus, and molecular interaction energy functions of LCA and HDCA. Surface pressure unit is expressed in mJ/m^2^ instead of the commonly applied mN/m.

The resistance of molecular films to compression (e.g., elasticity) is commonly characterized by the compressibility modulus (*K*), given as *K* = −*A*(*dπ*/*dA*)_*T*_, where *A* is the area per molecules, *π* is surface pressure in the films, and *T* is temperature. An increase in compressibility modulus reflects an increasing resistance of the film against compression and shows its gradual transition from fluid-like to solid-like behaviour. A decrease in compression modulus reflects the reversed process. Thus, maxima or minima of compressibility curves evidence the changes in orientation or aggregation state of molecules in the monolayer seen also as second- or first-order phase transitions in the compression isotherms. Representative compressibility modulus curves are plotted in blue in Figure 6 for the LCA and HDCA films. The maximum of *K*, in general, reflects some kind of configurational transition in the monolayer structure [39]: the films can either collapse or condense or undergo orientational ordering [38, 39]. For the BA films represented by LCA and HDCA, the maximum of *K* is observed at the inflection point of the isotherms (orientational ordering) marked with black vertical lines. Collapse and condensation are clearly not the case in the BA isotherms at the maximum of *K*, because at collapse, further compression would not allow for systematic and reproducible *π*-*A* functions and at condensation (first-order phase transition) a near horizontal plateau region should be found. However, orientational ordering can occur gradually, allowing for a gradual decrease in the slope after an identifiable inflection point observed in the isotherms. After the maximum, *K* decreases until the start of hydrophobic condensation, where it eventually drops to zero according to the abrupt change in the state of the film at the beginning of first-order phase transition.

The low stability of the BA films does not allow, unfortunately, the complete thermodynamic analysis of the compression isotherms. Nevertheless, we attempted to calculate the molecular interaction energies keeping in mind that the results are only indicative and that the actual values must be handled with caution. We calculated the molecular interaction energy parameters (J/molecule) in terms of the compression energy transmitted to the films along the compression isotherms by multiplying the values of surface pressure (mJ/m^2^) and corresponding molecular area values (Å^2^/molecule). The order of magnitude of this parameter (10^−21^ J = zJ) coincides with that of the Hamaker constants [55]. The green lines in Figure 6 are examples of the molecular interaction energy versus molecular area functions obtained for the LCA and HDCA films. These functions can be interpreted in the way that any change in their trend indicates a change in resistance of the film to compression force; that is, the points of slope change reveal the occurrence of molecular association. The values of the molecular interaction energies at these critical points are characteristic of the cohesion (association) energies of these molecules in two dimensions. The maximum of cohesion is seen at the beginning of the linear part of the isotherms (marked with black vertical lines), which represents the initiation of H-bonding between the reoriented molecules in the interface as discussed in Section 3.2. Chemically, only two types of interaction are plausible between BA molecules: H-bonding and hydrophobic interaction. Hydrophobic interaction is not specific and it can occur between any large amounts of molecules leading to condensation, which is a first-order phase transition with a nearly horizontal plateau in the isotherms. The linear parts in the BA isotherms are clearly not horizontal and this rules out hydrophobic interaction. The cohesion energies for hydrophobic aggregation are calculated at the start of condensation regions (marked with black vertical lines). The latter values are a bit but systematically lower than the H-bonding energies; the films can be compressed to lower molecular area values before aggregation occurs. Different pathways of condensation can lead to the coexistence of either liquid expanded and liquid condensed monomolecular phases or liquid expanded and its collapsed multilayer domains [52], but both reflect the maximum of compression energy resisted by the molecules just before complete condensation begins.

The molecular energies of H-bonding and hydrophobic interaction at air/water interface for the studied BAs are collected in Table 2. The values from the analysis of the compression isotherms harmonize with those obtained by Exerowa [56] for lateral interaction energies between phospholipid molecules in lipid bilayers (13 and 12 zJ for DMPC and DPPC) and by Dynarowicz and coauthors [29] for the interaction between stearic acid and 1-octadecanol in Langmuir monolayer (19.25 zJ). The interaction energies connected with the hydrophobic association of BAs decrease from 15.17 to 7.77 zJ (from LCA to UDCA) following approximately the order of increasing values of CMC (data in Table 1). The calculations are based on the molecular amounts of BAs spread on the surface and do not account for dissolution. If the dissolution of BAs had been evaluated, the results for corrected molecular areas would decrease less strongly. Although the area per molecule values are somewhat disturbed by dissolution, the surface pressure values are reliable, because *π* is the intensive property of all actual films. The interaction energies calculated at the end of orientational ordering (the start of the linear part of the isotherms), probably characterizing H-bonding, follow the same order, but the values are systematically higher compared to that of hydrophobic interaction. For the CnA film, relatively low hydrophobic interaction energy was obtained (9.5 zJ), yet close to the values obtained for phospholipids [29]. This can also be due to the disagreement between the formal and real molecular area values. CnA has only one hydrophilic moiety, the carboxylic group, which may not be fully efficient in anchoring the steroidal skeleton to the aqueous subphase and, so, the appearance of aggregated domains can be supposed already at the spreading. Poša determined the hydrophobicity of sodium salts of CA, CDCA, DCA, HDCA, and UDCA in chromatographic experiments [57] and the change in the chromatographic retention index values (*R*_M0_) follows in principle the order observed by us for the energies of association due to hydrophobic interaction in the Langmuir trough experiments. The interaction energies decrease with decreasing* R*_M0_, that is, increasing hydrophilicity.

## 4. Conclusions

Langmuir film study of hydroxy- and oxosubstituted bile acids was performed in order to get more insight into the aggregation/association behavior of these pharmacologically important molecules. Compared to bulk association studies, two-dimensional experiments have the advantage that the solute-solvent interactions are mostly excluded (except for the hydration of anchoring hydrophilic moieties) and the information obtained reflects more closely the intrinsic molecular association property of BAs.

The analysis of the compression isotherms measured on aqueous subphase of 3 M NaCl solution at pH 1.2 allowed to characterize molecular interactions in the film induced by decreasing area accessible for molecules. Principally different behavior was observed for the BAs with different hydrophilicity. Overall, the two-dimensional association behavior of BA molecules supports the following mechanism of pressure-forced association: formation of dimers via H-bonding followed by secondary aggregation via hydrophobic interaction. The less hydrophilic BAs (LCA, DCA, CDCA, HDCA, and UDCA) can associate by H-bonding and then by hydrophobic interactions. The most hydrophilic BAs (3DHCA, DHDCA, and DHCA) are unable to form either type of associates in floating film. BAs with intermediate hydrophilicity (3DHDCA, HCA, and CA) exploit the H-bonding possibility for association, but not the hydrophobic interaction.

The Langmuir trough experiments performed systematically on the series of bile acids and derivatives can be used to select appropriate molecules for specific aims, such as drug delivery vehicles or drug uptake enhancers. As a first approximation, the best candidates for incorporating into the cell membranes without solubilizing them should be found among those exhibiting minimized hydrophobic aggregation yet capable of H-bonding in the floating monolayers. The analysis of the compression isotherms allowed us to identify the sequence of H-bonding and hydrophobic interaction in BA association, while their temporal separation is not possible in any bulk experiments. We must note, however, that our findings cannot be regarded as a direct indication of the association mechanism of BA molecules in aqueous solutions.

## Supplementary Material

The pictures of spread LCA and DCA films on pure water and on 3 M NaCl solution (pH = 1.2) subphases were taken during 60 minutes. Selected pictures were used to create the movie showing the decrease in the film areas in time, relative to each other.
Left upper plane: LCA on pure water
Right upper plane: DCA on pure water
Left lower plane: LCA on NaCl solution
Right lower plane: DCA on NaCl solution

## Figures and Tables

**Table 1:** Solubility and CMC data of bile acids and their oxoderivatives taken from the literature.

Bile acids and oxoderivatives	Structure	Solubility mmol/L pH3, 25°C	CMC mmol/L Na-salt, 25°C
		References [47–50]	
CnA—cholanic acid 5*β*-cholanoic acid	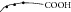	No data	No data

LCA—lithocholic acid 3-hydroxy-5*β*-cholanoic acid	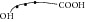	0.00005	1

DCA—deoxycholic acid 3*α*,12*α*-dihydroxy-5*β*-cholanoic acid	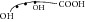	0.028	2–10

CDCA—chenodeoxycholic acid 3*α*,7*α*-dihydroxy-5*β*-cholanoic acid	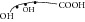	0.027	4–9

HDCA—hyodeoxycholic acid 3*α*,6*α*-dihydroxy-5*β*-cholanoic acid	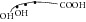	0.015	6–14

UDCA—ursodeoxycholic acid 3*α*,7*β*-dihydroxy-5*β*-cholanoic acid	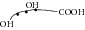	0.009	7–19

3DHDCA—3-dehydrodeoxycholic acid 3-oxo-12*α*-hydroxy-5*β*-cholanoic acid	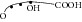	No data	17

DHDCA—dehydrodeoxycholic acid 3,12-dioxo-5*β*-cholanoic acid	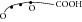	No data	51

CA—cholic acid 3*α*,7*α*,12*α*-trihydroxy-5*β*-cholanoic acid	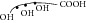	0.273	6–18

HCA—hyocholic acid 3*α*,6*α*,7*α*-trihydroxy-5*β*-cholanoic acid	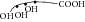	0.045	8–17

3DHCA—3-dehydrocholic acid 3-oxo-7*α*,12*α*-dihydroxy-5*β*-cholanoic acid	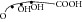	No data	98

DHCA—dehydrocholic acid 3,7,12-trioxo-5*β*-cholanoic acid	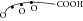	No data	130

**Table 2:** Molecular energy values calculated as (*π* · *A*) at characteristic points of the *π*-*A* isotherms as discussed in the text.

Bile acids	Energy of association, zJ/molecule	
	Hydrophobic	H-bonding
CnA	9.5	—
LCA	15.17	15.7
CDCA	13.1	17.59
DCA	11.9	14.2
HDCA	7.4	10.2
UDCA	7.77	9.7

## References

[1] Enhsen A., Kramer W., Wess G. (1998). Bile acids in drug discovery. *Drug Discovery Today*.

[2] Wiedmann T. S., Kamel L. (2002). Examination of the solubilization of drugs by bile salt micelles. *Journal of Pharmaceutical Sciences*.

[3] Calabresi M., Andreozzi P., La Mesa C. (2007). Supra-molecular association and polymorphic behaviour in systems containing bile acid salts. *Molecules*.

[4] Holm R., Müllertz A., Mu H. (2013). Bile salts and their importance for drug absorption. *International Journal of Pharmaceutics*.

[5] Swenson E. C., Curatolo W. J. (1992). Intestinal permeability enhancement for proteins, peptides and other polar drugs: mechanisms and potential toxicity. *Advanced Drug Delivery Reviews*.

[6] Reis S., Moutinho C. G., Matos C., de Castro B., Gameiro P., Lima J. L. F. C. (2004). Noninvasive methods to determine the critical micelle concentration of some bile acid salts. *Analytical Biochemistry*.

[7] Seret A., Bahri M.-A. (2009). The CMC-like behaviour of bile salts as probed by photoexcited Rose Bengal. *Colloids and Surfaces A: Physicochemical and Engineering Aspects*.

[8] Small D. M., Nair P. P., Kritchevsky D. (1971). The physical chemistry of cholanic acids. *The Bile Salts: Chemistry, Physiology, and Methabolism*.

[9] Warren D. B., Chalmers D. K., Hutchison K., Dang W., Pouton C. W. (2006). Molecular dynamics simulations of spontaneous bile salt aggregation. *Colloids and Surfaces A: Physicochemical and Engineering Aspects*.

[10] Kawamura H., Murata Y., Yamaguchi T., Igimi H., Tanaka M., Sugihara G., Kratohvil J. P. (1989). Spin-label studies of bile salt micelles. *Journal of Physical Chemistry*.

[11] D'Alagni M., D'Archivio A. A., Galantini L., Giglio E. (1997). Structural study of the micellar aggregates of sodium chenodeoxycholate and sodium deoxycholate. *Langmuir*.

[12] Jójárt B., Viskolcz B., Poša M., Fejer S. N. (2014). Global optimization of cholic acid aggregates. *The Journal of Chemical Physics*.

[13] Funasaki N., Fukuba M., Hattori T., Ishikawa S., Okuno T., Hirota S. (2006). Micelle formation of bile salts and zwitterionic derivative as studied by two-dimensional NMR spectroscopy. *Chemistry and Physics of Lipids*.

[14] Garidel P., Hildebrand A., Neubert R., Blume A. (2000). Thermodynamic characterization of bile salt aggregation as a function of temperature and ionic strength using isothermal titration calorimetry. *Langmuir*.

[15] Garidel P., Hildebrand A., Knauf K., Blume A. (2007). Membranolytic activity of bile salts: Influence of biological membrane properties and composition. *Molecules*.

[16] Yang L., Zhang H., Mikov M., Tucker I. G. (2009). Physicochemical and biological characterization of monoketocholic acid, a novel permeability enhancer. *Molecular Pharmaceutics*.

[17] Zhang Q., Ma X., Ward A. (2007). Designing facial amphiphiles for the stabilization of integral membrane proteins. *Angewandte Chemie*.

[18] Travaglini L., D'Annibale A., Schillén K., Olsson U., Sennato S., Pavel N. V., Galantini L. (2012). Amino acid-bile acid based molecules: extremely narrow surfactant nanotubes formed by a phenylalanine-substituted cholic acid. *Chemical Communications*.

[19] Dynarowicz-Ła̧tka P., Kita K. (1999). Molecular interaction in mixed monolayers at the air/water interface. *Advances in Colloid and Interface Science*.

[20] Möhwald H., Lipowsky R., Sackmann E. (1995). Phospholipid monolayers. *Handbook of Biological Physics*.

[21] Vollhardt D., Fainerman V. B. (2006). Progress in characterization of Langmuir monolayers by consideration of compressibility. *Advances in Colloid and Interface Science*.

[22] Wang L., Jacobi S., Sun J., Overs M., Fuchs H., Schaefer H. J., Zhang X., Shen J., Chi L. (2005). Anisotropic aggregation and phase transition in Langmuir monolayers of methyl/ethyl esters of 2,3-dihydroxy fatty acids. *Journal of Colloid and Interface Science*.

[23] Kaganer V. M., Möhwald H., Dutta P. (1999). Structure and phase transitions in Langmuir monolayers. *Reviews of Modern Physics*.

[24] Joly M. (1950). General theory of the structure, transformations and mechanical properties of monolayers. *Journal of Colloid Science*.

[25] Hedge D. G. (1957). An equation of state for lecithin monolayers. *Journal of Colloid Science*.

[26] Steinbach H., Sucker C. (1980). Structure of association in surface films. *Advances in Colloid and Interface Science*.

[27] Baldyga D. D., Dluhy R. A. (1998). On the use of deuterated phospholipids for infrared spectroscopic studies of monomolecular films: a thermodynamic analysis of single and binary component phospholipid monolayers. *Chemistry and Physics of Lipids*.

[28] Schmid F., Stadler C., Lange H. (1999). Theoretical modeling of Langmuir monolayers. *Colloids and Surfaces A: Physicochemical and Engineering Aspects*.

[29] Dynarowicz P., Jawień W., Miñones Trillo J., Vila Romeu N., Sanchez-Caballero C. V., Jado E. I., Mouzo O. C. (1995). Molecular interaction in mixed spread films at the water air interface. *Colloids and Surfaces A: Physicochemical and Engineering Aspects*.

[30] Andrade C. A. S., Santos-Magalhães N. S., de Melo C. P. (2006). Thermodynamic characterization of the prevailing molecular interactions in mixed floating monolayers of phospholipids and usnic acid. *Journal of Colloid and Interface Science*.

[31] Ha̧c-Wydro K., Dynarowicz-Ła̧tka P., Grzybowska J., Borowski E. (2005). Interactions of Amphotericin B derivative of low toxicity with biological membrane components—the Langmuir monolayer approach. *Biophysical Chemistry*.

[32] Fahey D. A., Carey M. C., Donovan J. M. (1995). Bile acid/phosphatidylcholine interactions in mixed monomolecular layers: differences in condensation effects but not interfacial orientation between hydrophobic and hydrophilic bile acid species. *Biochemistry*.

[33] Gaines G. L. (1966). *Insoluble Monolayers at Liquid-Gas Interfaces*.

[34] Carey M. C., Danielsson H., Sjövall J. (1985). Physical chemical properties of bile acids and their salts. *New Comprehensive Biochemistry*.

[35] Ekwall P., Ekholm R., Norman A. (1957). Surface balance studies of bile acid monolayers, I. Cholanic and Glycocholanic monolayers. *Acta Chemica Scandinavica*.

[36] Ekwall P., Ekholm R., Norman A. (1957). Surface balance studies of bile acid monolayers, II. Monolayers of lithocholic acids. *Acta Chemica Scandinavica*.

[37] Kauffman J. M., Pellicciari R., Carey M. C. (2005). Interfacial properties of most monofluorinated bile acids deviate markedly from the natural congeners: studies with the Langmuir-Pockels surface balance. *The Journal of Lipid Research*.

[38] Gálvez-Ruiz M. J., Cabrerizo-Vílchez M. A. (1991). Structural and stability analysis of monolayers of some bile acids at the air-aqueous solution interface. *Colloids and Surfaces*.

[39] Messina P. V., Prieto G., Ruso J. M., Fernández-Leyes M. D., Schulz P. C., Sarmiento F. (2010). Thermodynamic and elastic fluctuation analysis of langmuir mixed monolayers composed by dehydrocholic acid (HDHC) and didodecyldimethylammonium bromide (DDAB). *Colloids and Surfaces B: Biointerfaces*.

[40] Matsuoka K., Takagi K., Honda C. (2013). Micelle formation of sodium hyodeoxycholate. *Chemistry and Physics of Lipids*.

[41] Nagadome S., Suzuki N. S., Mine Y., Yamaguchi T., Nakahara H., Shibata O., Chang C.-H., Sugihara G. (2007). Monolayers (Langmuir films) behavior of multi-component systems composed of a bile acid with different sterols and with their 1:1 mixtures. *Colloids and Surfaces B: Biointerfaces*.

[42] Kuhajda K., Kevrešan S., Kandrač J., Fawcett J. P., Mikov M., Mikov M., Fawcett J. P. (2007). Chemical and metabolic transformations of selected bile acids. *Bile Acids: Chemistry, Biosynthesis, Analysis, Chemical and Metabolic Transformations and Pharmacology*.

[43] Tserng K. Y. (1978). A convenient synthesis of 3-keto bile acids by selective oxidation of bile acids with silver carbonate-Celite. *Journal of Lipid Research*.

[44] Fieser L. F., Rajagopalan S. (1950). Oxidation of steroids. III. Selective oxidations and acylations in the bile acid series. *Journal of the American Chemical Society*.

[45] Moss G. P. (1982). IUPAC-IUB Joint Commission on Biochemical Nomenclature (JCBN). The nomenclature of steroids. Recommendations 1989. *European Journal of Biochemistry*.

[46] Hofmann A. F., Sjovall J., Kurz G., Radominska A., Schteingart C. D., Tint G. S., Vlahcevic Z. R., Setchell K. D. R. (1992). A proposed nomenclature for bile acids. *Journal of Lipid Research*.

[47] Poša M. (2011). QSPR study of the effect of steroidal hydroxy and oxo substituents on the critical micellar concentration of bile acids. *Steroids*.

[48] Hofmann A. F., Roda A. (1984). Physicochemical properties of bile acids and their relationship to biological properties: an overview of the problem. *Journal of Lipid Research*.

[49] Fini A., Feroci G., Roda A. (2002). Acidity in bile acid systems. *Polyhedron*.

[50] Simonović B. R., Momirović M. (1997). Determination of critical micelle concentration of bile acid alts by micro-calorimetric titration. *Mikrochimica Acta*.

[51] Johnson M. J., Majmudar C., Skolimowski J. J., Majda M. (2001). Critical temperature and LE/G phase transitions in monolayer films of the amphiphilic TEMPO derivatives at the air/water interface. *The Journal of Physical Chemistry B*.

[52] Devries C. A., Haycraft J. J., Han Q., Noor-E-Ain F., Raible J., Dussault P. H., Eckhardt C. J. (2011). Reversible collapse of the Langmuir films of a series of triphenylsilyl ether-terminated amphiphiles. *Thin Solid Films*.

[53] Kellner B. M. J., Cadenhead D. A. (1978). Monolayer studies of hydroxyhexadecanoic acids. *Journal of Colloid and Interface Science*.

[54] Bonosi F., Romanelli M., Martini G., Ricciardi G., Lelj F. (1994). Monolayer and multilayer films of differently substituted metal tetraazaannulene complexes. *Thin Solid Films*.

[55] Visser J. (1972). On Hamaker constants: a comparison between Hamaker constants and Lifshitz-van der Waals constants. *Advances in Colloid and Interface Science*.

[56] Exerowa D. (2002). Chain-melting phase transition and short-range molecular interactions in phospholipid foam bilayers. *Advances in Colloid and Interface Science*.

[57] Poša M., de Azevedo Calderon L. (2012). Chromatographic retention parameters as molecular descriptors for lipophilicity in QSA(P)R studies of bile acid. *Chromatography—The Most Versatile Method of Chemical Analysis*.

